# Editorial: Nanotechnology in Cardiovascular Regenerative Medicine

**DOI:** 10.3389/fbioe.2020.608844

**Published:** 2020-10-23

**Authors:** Wenguo Cui, Aijun Wang, Chao Zhao, Wuqiang Zhu

**Affiliations:** ^1^Ruijin Hospital, Shanghai Jiao Tong University School of Medicine, Shanghai, China; ^2^Department of Surgery and Biomedical Engineering, University of California, Davis, Davis, CA, United States; ^3^Department of Chemical and Biological Engineering, University of Alabama at Tuscaloosa, Tuscaloosa, AL, United States; ^4^Department of Cardiovascular Diseases, Physiology and Biomedical Engineering, Center of Regenerative Medicine, Mayo Clinic, Scottsdale, AZ, United States

**Keywords:** nanotechnology, cardiovascular, regenerative medicine, nanoparticle, tissue engineering, heart failure

Due to their unique properties, nanomaterials can provide novel opportunities in cardiovascular tissue engineering. Thus far, nanomaterial and nanotechnologies have been extensively tested in various fields of cardiovascular tissue engineering, including drug delivery, engineered cardiac muscle, and vascular grafts. In this special issue, we have focused on various aspects of nanotechnologies (and nanomaterials) relevant to cardiovascular regenerative medicine.

## Nanotechnologies in Vascular and Microvascular Regeneration and Repair

Many types of vascular diseases lead to stenosis and reduced blood perfusion. The mainstay treatments include thrombolytic medications and surgical procedures to re-establish the blood flow, such as angioplasty and bypass surgery with autologous or artificial vessel grafts. Nanotechnologies possess unique value in treating vascular disease. Novel nanomaterials may deliver drugs to lesion sites after intravascular administration. In this special issue, several studies demonstrated that novel nanotechnologies promotes angiogenesis from vascular cells and accelerate wound healing.

The stromal vascular fraction (SVF) is a heterogeneous population of cells that are derived from subcutaneous fat. Hu et al. reported that aligned nanofibrillar scaffolds promote cell attachment and enhance the secretion of VEGF from attached SVF cells. Transplantation of SVF-seeded nanomaterials improves blood perfusion recovery in a mouse model of peripheral arterial disease. Endothelial cells are the major component of the vascular structure. Activation of integrin signaling via hydrogel scaffold-mediated delivery of integrin ligands promotes endothelial cell (EC) proliferation and angiogenesis. First, Hao, Liu et al. reported that conjugation of LXW7, an integrin αvβ3 ligand, to the collagen backbone increases EC specific integrin binding sites on the collagen hydrogel. LXW7-treated collagen surface significantly improves cell attachment, proliferation, and survival of ECs under hypoxia conditions. In a mouse subcutaneous implantation model, LXW7-modified collagen hydrogel improves the engraftment of transplanted ECs and enhances vascular formation. Extracellular vesicles (EVs) modified biomaterials represent a new functional biomaterial and hold promise for tissue engineering and regenerative medicine applications. Hao, Swindell et al. further demonstrated that EVs from human placenta-derived mesenchymal stem cells (PMSCs) expressed integrin α4β1. Immobilizing the PMSC-EVs onto the electrospun extracellular matrix-mimicking scaffolds promotes EC migration and vascular sprouting in an *ex vivo* rat aortic ring assay. Zhou et al. reported that a copper sulfide nanoparticles-incorporated hyaluronic acid (CuS/HA) injectable hydrogel promotes wound healing in the rat skin wound model via promoting the expression of vascular endothelial growth factor (VEGF) and enhancing angiogenesis. This novel biomaterial holds promising potential for treating skin wounds.

Engineered vessel grafts represent a promising alternative to autografts and are frequently employed in the surgery clinics. Poly (styrene-block-butadieneblock-styrene) (SBS) is a kind of widely used thermoplastic elastomer with good mechanical properties and biocompatibility. Tan et al. demonstrated that synthesized anthracene-grafted SBS (SBS-An) promotes adhesion and proliferation of human umbilical vein endothelial cells (HUVECs) on a biomimetic elastic membrane with a switchable Janus structure. This approach may have great implication in vascular tissue engineering.

## Nanotechnologies in Myocardial Regeneration and Repair

Engineered heart tissue emerges as a promising approach for repairing the injured heart. However, the fabrication of a cardiac tissue with optimal biomechanical properties and high biocompatibility remains a challenge. Kumar et al. fabricated an aligned PCL-Gelatin coaxial nanofiber patch using human induced pluripotent stem cell-derived cardiomyocytes (hiPSC-CMs) and electrospinning. Nanofibers improve the maturation of hiPSC-CMs and their response to cardioactive drugs, and promote the formation of functional syncytium of these cardiac tissues. These biomimetic cardiac patches are potential models for “clinical trials in a dish” and for heart repair.

Nanomaterials carrying drugs are promising in enhancing the cardioprotective potential of drugs in patients with cardiovascular diseases. Deng et al. provided a comprehensive review of different types of nanomaterial-drug delivery systems and their applications in cardiovascular imaging and the treatment of various cardiovascular diseases. Nanomaterials have been shown to provide sustained exposure precisely to the ischemic myocardium. Fan et al. systemically reviewed the recent advances and challenges of different types of nanoparticles loaded with agents for the treatment of ischemic heart disease. Liao et al. summarized the composition, advantages, and disadvantages of different injectable hydrogels in the diagnosis and treatment of cardiovascular diseases. Angioplasty with an intra-vessel stent is an effective method for treating coronary heart disease. However, thrombosis and restenosis are the two major issues that often lead to device failure. Rao et al. provided a comprehensive review of the nanotechnologies on generating NO-leasing stents and the NO-producing strategies in anti-thrombosis and restenosis treatments.

Molecular imaging (MOI) or biomarkers has been commonly used in the diagnosis of cardiovascular diseases. However, sensitivity, specificity, and accuracy of the assay are still challenging for the early stage of cardiovascular diseases. Shi et al. nicely summarized the cardiac biomarkers and imaging techniques that are currently used for CVD diagnosis, and discussed the applications of various nanotechnologies on improving the value of cardiac immunoassays and molecular imaging in the detecting early stage of cardiovascular diseases.

## Nanotechnologies in the Treatment of Cardiovascular Inflammation

Inflammation contributes to the pathogenesis of vessel diseases such as arteriosclerosis and restenosis. It is not clear whether the morphology of vascular ECs has any impact on the activation of monocytes and other inflammatory cells. Liang et al. showed that elongated ECs cultured on poly-(dimethyl siloxane) membrane surface with microgrooves significantly suppressed the activation of the monocytes in co-culture, in comparison to the ECs with a cobblestone shape. Further investigation demonstrated that EC morphology can regulate the response of inflammatory cells through the secretion of specific miRNAs. These data provides a basis for the design and the optimization of biomaterials for vascular tissue engineering.

The objective of cardiovascular immunotherapy is to develop approaches that suppress excessive inflammatory responses. Yi et al. engineered an injectable filamentous hydrogel depot (FM-depot) for low dosage, sustained delivery of anti-inflammatory nanocarriers. Specifically, the bioactive form of vitamin D (aVD; 1, 25-Dihydroxyvitamin D3), which inhibits pro-inflammatory transcription factor NF-kB via the intracellular nuclear hormone receptor vitamin D receptor (VDR), was stably loaded into poly(ethylene glycol)-block-poly(propylene sulfide) (PEG-b-PPS) filomicelles. Following crosslinking with multi-arm PEG for in situ gelation, aVD-loaded FM-depots maintained high levels of Foxp3+ Tregs in both lymphoid organs and atherosclerotic lesions for weeks following a single subcutaneous injection into ApoE^−/−^ mice. These data suggested that nanomaterial-based slow release of anti-inflammatory chemicals may be effective to enhance cardiovascular immunotherapy.

Deferoxamine (DFO) has long been used as an FDA-approved iron chelator. Conjugation of DFO with polymers improves their plasma half-life and reduces toxicity when administered intravenously. However, the blood interactions and antioxidation of the DFO-conjugates and the mechanisms underlying these outcomes remain to be elucidated. Sun et al. reported that alginate-DFO conjugates (ADs) inhibit the intrinsic pathways in the process of coagulation, and activate the complements C3a and C5a Ds in a dose-dependent manner through an alternative pathway. These data implicated that Ads induce the cross-talking among coagulation, complement and platelet.

The editors are grateful to the authors who contributed their original research articles and invited reviews to this Special Issue. These articles provided an excellent outline of the current status of nanotechnologies in cardiovascular regenerative medicine.

## Author Contributions

All authors listed have made a substantial, direct and intellectual contribution to the work, and approved it for publication.

## Conflict of Interest

The authors declare that the research was conducted in the absence of any commercial or financial relationships that could be construed as a potential conflict of interest.

